# Second trimester uterine incarceration in monochorionic twins requiring fetal intervention

**DOI:** 10.1002/pmf2.70066

**Published:** 2025-07-02

**Authors:** Courtney T. Connolly, Shriddha Nayak, Lauren N. Meiss, Michelle L. Kush, Jennifer Kearney, Michael M. Aziz, Erin Gomez, Ahmet A. Baschat, Jena L. Miller, Mara Rosner

**Affiliations:** ^1^ Johns Hopkins Center for Fetal Therapy Department of Gynecology and Obstetrics Johns Hopkins University School of Medicine Baltimore Maryland USA; ^2^ Division of Maternal Fetal Medicine Department of Gynecology and Obstetrics Johns Hopkins University School of Medicine Baltimore Maryland USA; ^3^ Division of Maternal Fetal Medicine Department of Obstetrics and Gynecology Allegheny Health Network Pittsburgh Pennsylvania USA; ^4^ The Russell H. Morgan Department of Radiology and Radiological Science Johns Hopkins University School of Medicine Baltimore Maryland USA

## Abstract

**Background:**

Uterine incarceration is a rare occurrence characterized by entrapment of the gravid uterine fundus between the sacral promontory and pubic symphysis. Mid‐trimester presentation has additional obstetric and surgical significance.

**Case:**

We report a patient with second‐trimester incarcerated uterus in a complicated monochorionic diamniotic twin pregnancy with a suspected bicornuate uterus. Uterine incarceration distorted the ultrasound appearance of fetal and placental location. Reduction of the incarcerated fundus was required to restore the abdominopelvic and intrauterine anatomy prior to fetal intervention. Selective reduction of the growth‐restricted, anomalous fetus was then successfully performed.

**Conclusion:**

Uterine incarceration distorts the maternal‐pelvic anatomy as well as the perceived location of intrauterine contents. Recognition of altered intrauterine landmarks is critical when obstetric or surgical management is indicated. Our case highlights the distortion of intrauterine contents associated with uterine incarceration and the potential impact on obstetric and surgical management.

## 1 INTRODUCTION

Incarceration of the gravid uterus occurs when the growing fundus becomes wedged between the sacral promontory and the pubic symphysis. It affects 1 in 3,000 to 10,000 pregnancies, primarily in first‐trimester patients with a retroverted or retroflexed uterus [1, 2]. When uterine incarceration persists, there is marked stretching of the cervix and lower segment anteriorly, and the growing fundus remains deeply impacted in the posterior cul‐de‐sac. This can cause constipation, urinary obstruction, and pelvic vein thrombosis [2]. Uterine incarceration in the first trimester may be managed expectantly with some cases undergoing spontaneous resolution as the fundus grows [3]. However, manual reduction before 20 weeks is recommended in cases of uterine incarceration that persist into the second trimester due to low rates of spontaneous resolution as well as risks. Obstetric complications including prelabor rupture of membranes, preterm delivery, labor dystocia, and uterine rupture have been reported, as well as surgical morbidity at the time of cesarean delivery (CD) including inadvertent cystotomy or cervical incision [1, 2, 3, 4]. We present a case of second‐trimester uterine incarceration in a patient with a uterine anomaly and complicated monochorionic diamniotic twins that highlights the importance of recognizing uterine incarceration in the setting of presurgical planning and the potential impact of uterine restitution on fetal well‐being.

### 1.1 Case

A 37‐year‐old G4P1021 with a suspected bicornuate uterus and monochorionic diamniotic twins in the right horn was evaluated at 17 weeks 5 days for suspected Twin to Twin Transfusion Syndrome (TTTS), selective fetal growth restriction (sFGR), and a large omphalocele in the growth‐restricted fetus (Fetus 2). She had a history of a full‐term CD for malpresentation at which time a bicornuate uterus was suspected.

At the 12‐week ultrasound, monochorionicity was confirmed along with a large omphalocele of Fetus 2, and she underwent genetic counseling. The patient opted to pursue chorionic villus sampling; however, it was unable to be performed given no safe access to the placenta could be identified. The patient desired to have information as soon as possible to make decisions regarding the management of the pregnancy and therefore opted to attempt an early amniocentesis at 15 weeks 1 day gestation. The sac of Fetus 2 was also inaccessible at that time, and a decision was made to perform amniocentesis only for Fetus 1 given presumed monozygosity. Microarray results were normal. The patient was subsequently referred for fetal therapy due to suspected TTTS at 17 weeks 0 days with polyhydramnios in Fetus 1 and oligohydramnios with absent end‐diastolic flow in Fetus 2.

**FIGURE 1:**
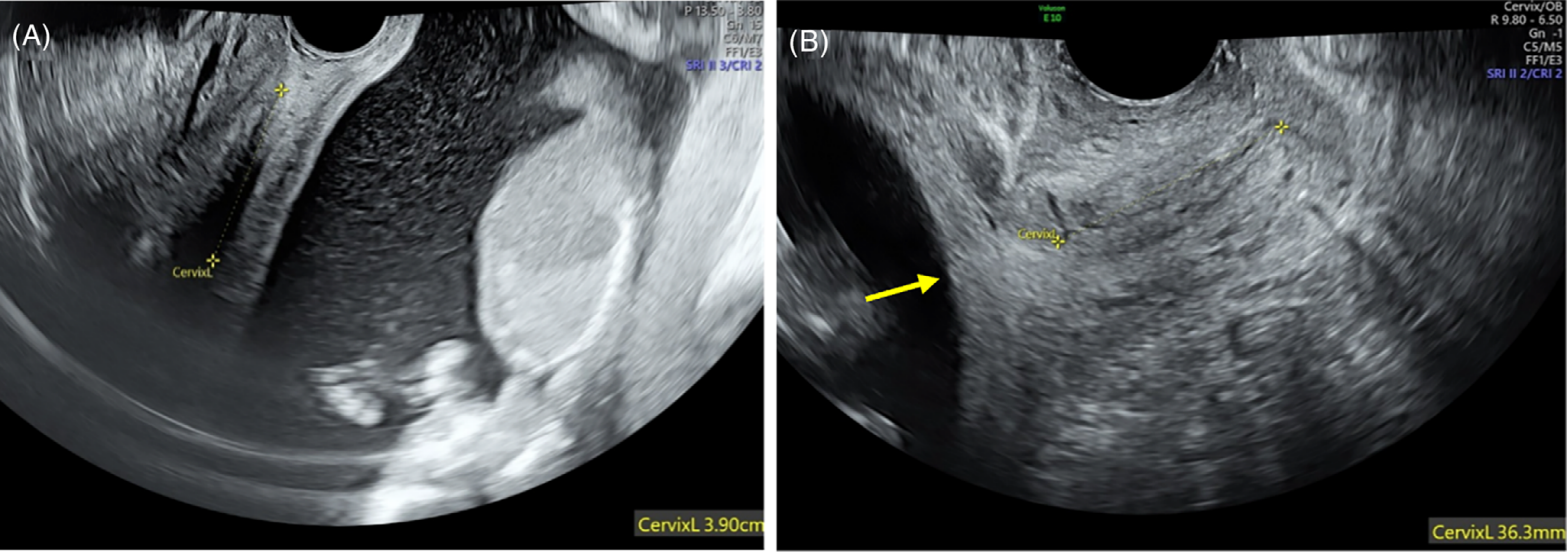
(A) Transvaginal cervical length at 17 weeks 5 days in the setting of uterine incarceration. Measurement of the cervical length is estimated; accurate cervical length was not possible as the internal os could not be visualized in continuity with the lower uterine segment due to uterine incarceration. (B) Transvaginal cervical length at 17 weeks 6 days immediately after successful uterine reduction demonstrates a normal cervical length with some redundancy of the lower uterine segment myometrium (arrow) proximal to the internal os.

Evaluation at our fetal therapy center was notable for isolated polyhydramnios of Fetus 1 and low but normal fluid for Fetus 2, not meeting TTTS criteria. Fetus 2 was noted to be deeply inferior and was only well‐visualized by transvaginal ultrasound which demonstrated that all biometric parameters, including the AC which did not include the omphalocele, were less than the first percentile with an EFW discordance of 25.4% between the twins. There was also intermittent absent and reversed end‐diastolic flow in the umbilical artery, meeting criteria for sFGR Type III [5]. The middle cerebral arteries and ductus venosus were normal for both fetuses. The placenta appeared to be posterior, and there was a myometrial band suspicious for a uterine synechiae in the left lower quadrant. Transvaginal ultrasound revealed a thin, elongated, anteriorly displaced cervix without visible continuity between the internal cervical os and the lower uterine segment, raising suspicion for uterine incarceration (Figure 1A). Vaginal exam was performed revealing a large posterior bulge in the vagina. Her only symptoms were abdominal distension and a lateral stretching sensation, which was typical for patients presenting with polyhydramnios or TTTS. Notably, she had no urinary symptoms, a more common presenting symptom of incarcerated uterus.

**FIGURE 2:**
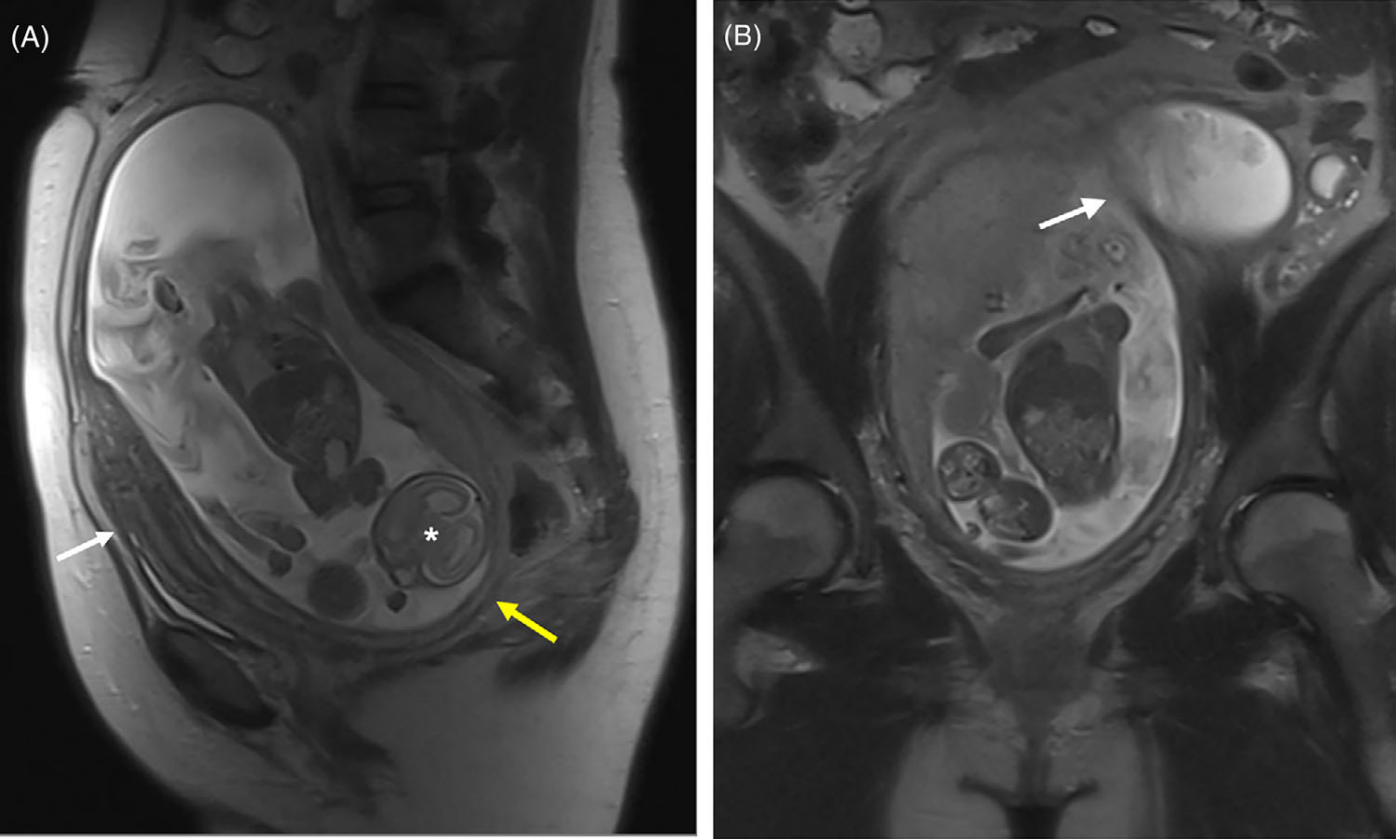
(A) Midline sagittal T2‐weighted MRI of the pelvis demonstrating incarcerated uterus with elongation and anterior displacement of the cervix (thin white arrow). The uterine fundus (thick yellow arrow) and the head of Fetus 2 (asterisk) are located in the presacral space. (B) Coronal T2‐weighted MR image of the pelvis demonstrating a T2 hypointense fibrous band at the uterine fundus (arrow).

Management options were discussed, and the patient opted for selective fetal reduction of the anomalous growth‐restricted Fetus 2. However, the sac of Fetus 2 could not be surgically accessed given its inferior location and the overlying maternal cervix. Magnetic resonance imaging (MRI) was performed as an adjunct to her ultrasound imaging secondary to uterine anomaly. MRI confirmed uterine incarceration with the fundus located in the presacral space, distention of the lower uterine segment, and elongation and anterior displacement of the cervix (Figure 2). The MRI also noted a left lateral intrauterine fibrous band, which likely reflected the suspected uterine anomaly.

**FIGURE 3:**
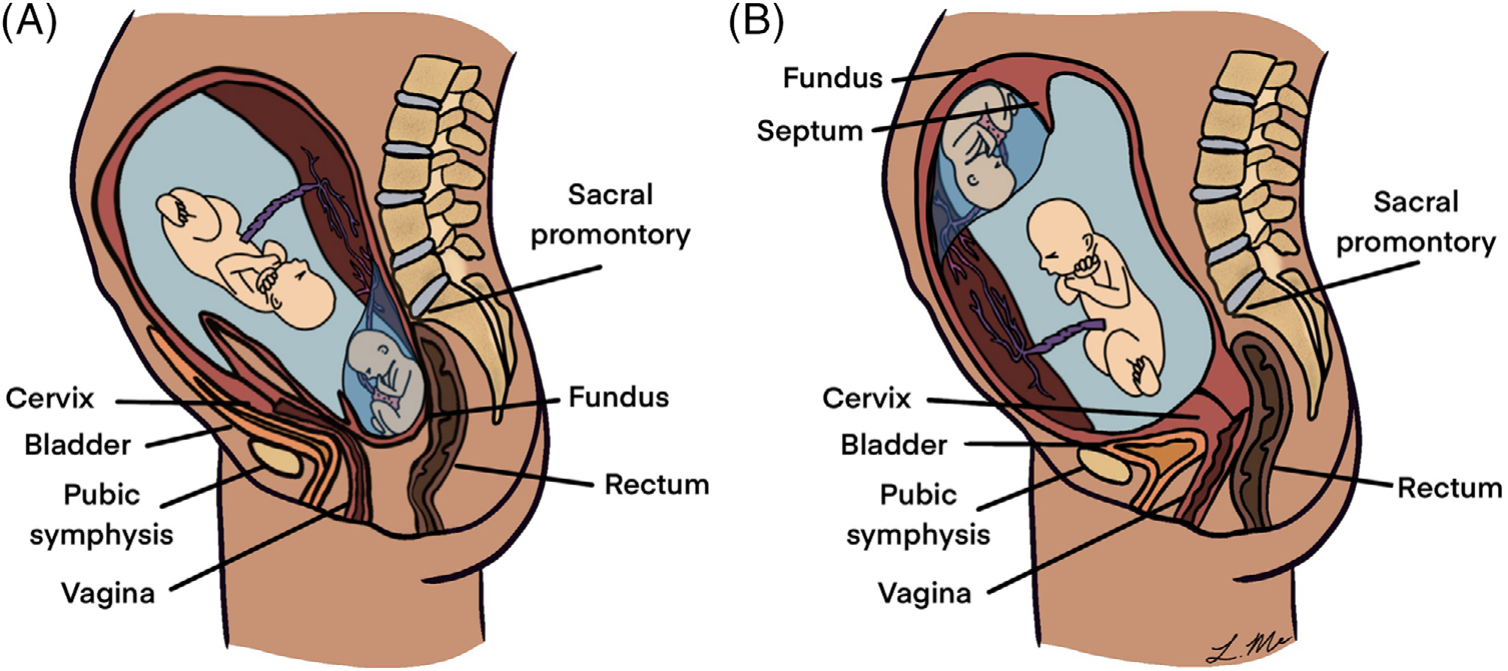
(A) Schematic of the abdominopelvic and intrauterine distortion in the setting of uterine incarceration. (B) Subsequent restitution of the abdominopelvic and intrauterine anatomy after resolution of uterine incarceration.

The patient underwent an uncomplicated manual vaginal reduction of her incarcerated uterus under spinal anesthesia in dorsal lithotomy at 17 weeks 6 days. Post‐procedure transvaginal ultrasound confirmed restoration of normal uterine position and cervical orientation (Figure 1b) with Fetus 2 superior in the uterine fundus and the previously posterior appearing placenta now clearly anterior (Figure 3). The fetal heart rates and Dopplers were reassuring, and she described immediate relief of her symptoms. Selective fetal reduction was delayed until the following day due to uterine irritability. The next day, at 18 weeks 0 days, Fetus 2 was noted to have low velocity, fixed reversed umbilical artery diastolic flow and absent fetal movement, and she underwent uncomplicated selective fetal reduction via microwave ablation of Fetus 2.

**FIGURE 4:**
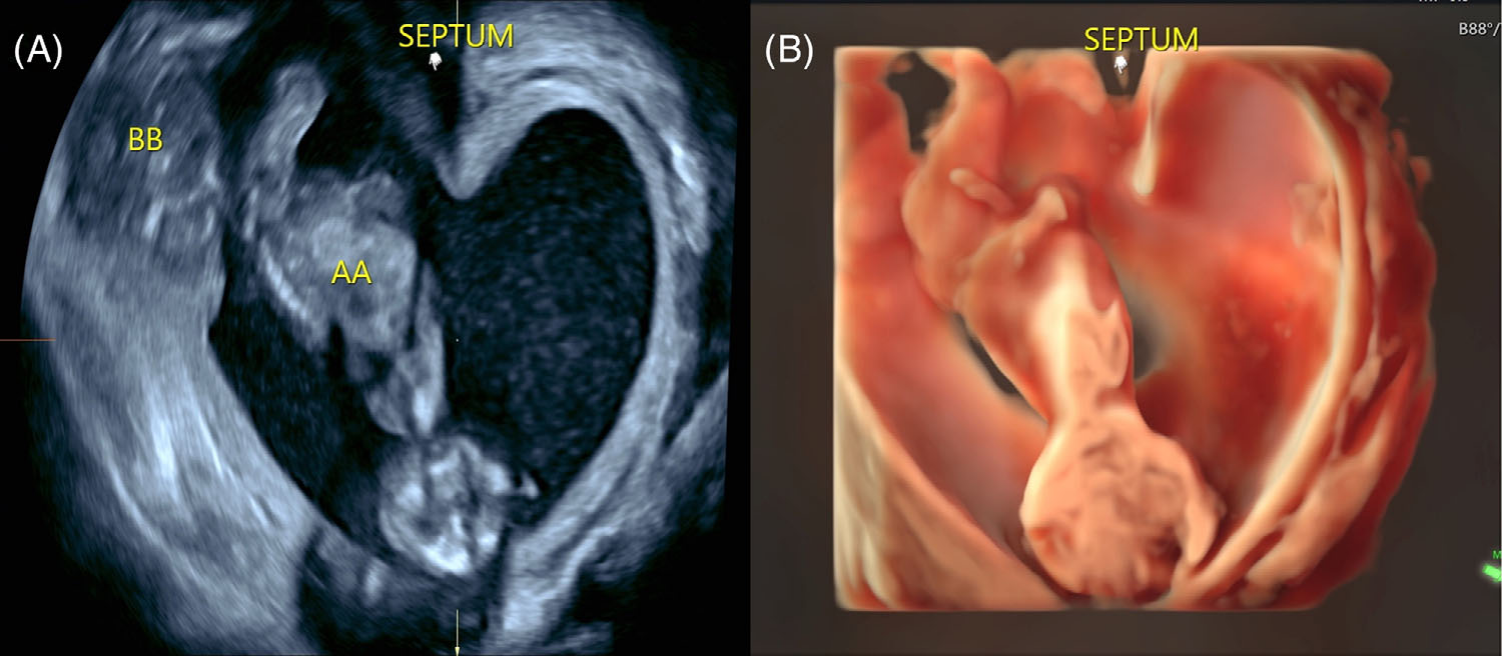
(A) Three‐dimensional multiplanar imaging of the uterine contour after reduction of the incarcerated uterus. A uterine septum is noted with indentation of the fundal uterine contour, consistent with her prior diagnosis of bicornuate uterus. Fetus 1 (labeled AA) is located on the right. Fetus 2 is labeled BB. (B) Three‐dimensional rendering of the same image emphasizing the heart‐shaped intrauterine cavity and ongoing fetus.

The next day Fetus 1 had normal activity and Dopplers with stable mild polyhydramnios and normal cervical length. Detailed ultrasound mapping of the internal uterine contour using multiplanar and 3‐dimensional (3D) rendering identified the previously suspected synechiae in the left lower quadrant to be superior‐fundal in location, consistent with her history of bicornuate uterus (Figure 4); postnatal confirmation of the uterine anomaly was recommended.

At 21 weeks, her cervical length was 21 mm, she declined cerclage, and vaginal progesterone was initiated. She presented with scant vaginal bleeding at 29 weeks 3 days and received betamethasone. She subsequently re‐presented at 30 weeks with vaginal bleeding, was found to be fully dilated, and underwent a repeat classical Cesarean section for breech presentation with an underdeveloped lower uterine segment. She delivered a viable male infant weighing 1370 grams with normal Apgar scores. NICU admission was uneventful, and he was discharged home in good condition.

## 2 DISCUSSION

Uterine incarceration is a rare finding affecting 1 in 3000 to 10,000 pregnancies in which an enlarging retroverted or retroflexed uterus becomes trapped between the sacral promontory and pubic symphysis. This can cause significant adverse outcomes both prenatally and intrapartum. Risk factors include conditions that may prevent a growing uterus from ascending out of the presacral area, including uterine anomalies, fibroids, adhesive disease, large ovarian cysts, an overhanging sacral promontory, and theoretically multifetal gestation [2, 3]. To our knowledge, this is only the second reported case of uterine incarceration in a multifetal gestation and the first case in which a fetal intervention was also performed. The previously reported case of uterine incarceration in a multifetal gestation was a triplet gestation at 9 weeks, which resolved with transvaginal pressure under epidural anesthesia [6].

Uterine incarceration may present acutely with severe urinary retention, or with only subtle signs, such as mild abdominal pain, lagging fundal height, and difficulty identifying the cervix on ultrasound or digital exam [3, 7]. Once identified, cases can be managed expectantly or with supportive care only in the first trimester due to high rates of spontaneous resolution. However, if incarceration persists into the second trimester, manual reduction is recommended to prevent adverse obstetric outcomes, such as labor dystocia, preterm delivery, uterine rupture, and unintended injury at the time of CD.

Our case specifically highlights the severity of both abdominopelvic and intrauterine anatomic distortion that occurs with second‐ or third‐trimester uterine incarceration and the importance of recognizing an incarcerated gravid uterus in any patient requiring surgical or obstetric intervention. If uterine incarceration goes unrecognized, a fundal placenta can be misdiagnosed as a low‐lying placenta or placenta previa, and fetal presentation can also be misidentified [3, 7]. Distorted anatomy at the time of CD can lead to inadvertent cervical, bladder, or vaginal injury and even cervical hysterectomy [2, 4, 7]. Restitution of our patient's incarcerated uterus demonstrated that the previously appearing posterior placenta was truly anterior, the previously inferior Fetus 2 was fundal, and the previously noted lower segment synechiae was likely representative of the uterine malformation (Figure 3). In retrospect, the inability to safely access Fetus 2 during attempts at both chorionic villus sampling and amniocentesis could have raised suspicion of an incarcerated uterus.

Incarcerated uterus can potentially compromise uteroplacental blood flow, likely due to compression of the uterine arteries, and has been associated with increased rates of fetal growth restriction and intrauterine fetal demise [2, 4]. In our case, we do not primarily attribute the sFGR to uterine incarceration as growth restriction is common both with omphalocele and in monochorionic gestations. However, we suspect that the acute change in the status of Fetus 2 observed on the day after the manual reduction may have been related to hemodynamic shifts in the setting of an already compromised fetus. We believe it is therefore reasonable to consider fetal monitoring after reduction of uterine incarceration, when clinically appropriate. The extent and type of monitoring should be individualized based on gestational age and relevant fetal conditions, keeping in mind the most significant hemodynamic alterations will likely occur within the first 24 h after reduction.

Some authors have suggested that manual reduction of an incarcerated uterus should only be performed prior to 20 weeks’ gestation due to increased risk of preterm labor and pregnancy loss [2]. In our case, it was not feasible to await spontaneous resolution due to the need to restore the anatomy to allow for surgical intervention. In cases where manual reduction fails, other techniques include inflating a Bakri balloon to 300cc in the vagina or more invasive interventions via colonoscopy or laparoscopic surgery have been described [1, 2, 3, 8]. Regardless of the technique, outcomes after second‐ and third‐trimester uterine incarceration are poorly understood due to the rarity and heterogeneity of the condition. Our patient developed a short cervix and preterm labor, resulting in preterm delivery. Although her course could also be attributed to monochorionic twins and the fetal procedure, uncomplicated selective fetal reduction in monochorionic twins is more commonly associated with a later gestational age at delivery. Future research is needed to ascertain the obstetric risks post‐reduction, as well as studying the changes in both uterine perfusion and fetal dopplers post‐reduction.

In conclusion, obstetricians should be aware of subtle symptoms, diagnostic clues, and anatomic distortion that can occur with uterine incarceration given its high risk of subsequent obstetric adverse outcomes. Awareness is critical to formulate individualized management strategies that may serve to minimize complications, particularly in the setting of planned fetal or obstetric procedures.
